# Dobutamine, Epinephrine, and Milrinone Accelerate Particle Transport Velocity in Murine Tracheal Epithelium via Ca^2+^ Release from Caffeine-Sensitive Internal Stores

**DOI:** 10.3390/cells14030228

**Published:** 2025-02-05

**Authors:** Götz Schmidt, Frederic Borchers, Sabrina Müller, Amir Ali Akbari, Fabian Edinger, Michael Sander, Christian Koch, Michael Henrich

**Affiliations:** 1 Department of Anesthesiology, Operative Intensive Care Medicine and Pain Therapy, Justus Liebig University Giessen, Rudolf-Buchheim-Strasse 7, 35392 Giessen, Germany; frederic-maximilian.borchers@med.uni-giessen.de (F.B.); sabrina.mueller@chiru.med.uni-giessen.de (S.M.); amir.ali-akbari@chiru.med.uni-giessen.de (A.A.A.); fabian.edinger@chiru.med.uni-giessen.de (F.E.); michael.sander@chiru.med.uni-giessen.de (M.S.); christian.koch@chiru.med.uni-giessen.de (C.K.); 2 Department of Anesthesiology, Intensive Care Medicine, Emergency Medicine, Vidia St. Vincentius-Clinic Karlsruhe gAG, 76135 Karlsruhe, Germany; michael.henrich@vincentius-ka.de

**Keywords:** tracheal epithelial cell, mucociliary clearance, ciliary beat frequency, perioperative, ciliary activity

## Abstract

Mucociliary clearance, the ability of the respiratory tract to protect the integrity of the airways through the mechanical removal of potentially harmful substances, is of enormous importance during intensive care treatment. The present study aimed to evaluate the influence of clinically relevant inotropic agents on mucociliary clearance. The particle transport velocity (PTV) of isolated murine tracheae was measured as a surrogate for mucociliary clearance in the presence of dobutamine, epinephrine, and milrinone. Inhibitory substances were applied to elucidate the signal transduction cascades and the value and origin of calcium ions which provoke alterations in mucociliary clearance function. Dobutamine, epinephrine, and milrinone increased the PTV in a dose-dependent manner with half maximal effective concentrations of 75.7 nM, 87.0 nM, and 13.7 µM, respectively. After the depletion of intracellular calcium stores, no increase in PTV was observed after administering any of the three inotropic agents. While dobutamine and epinephrine activated β-adrenergic receptors, epinephrine used both the phospholipase C (PLC) and protein kinase A (PKA) pathway to promote the release of intracellular Ca^2+^. However, dobutamine primarily acted on the PKA pathway, having only a minor influence on the PLC pathway. The induced changes in PTV following milrinone administration required both the PKA and PLC pathway, although the PKA pathway was responsible for most of the induced changes. In conclusion, the common inotropic agents dobutamine, epinephrine, and milrinone increase murine PTV in a concentration-dependent manner and ultimately release Ca^2+^ from intracellular calcium stores, suggesting the function of changes in mucociliary clearance in the respiratory tract.

## 1. Introduction

Inotropic agents are frequently administered during intensive care therapy and in the perioperative setting. These substances are used to improve cardiac contractility in various clinical conditions to achieve sufficient cardiac output and to ensure adequate tissue perfusion and oxygenation [1]. For example, inotropes are routinely used during cardiac surgery, and they form part of goal-directed hemodynamic therapy algorithms applied during non-cardiac surgery [2,3]. Inotropic agents are also administered in various presentations of cardiogenic shock or cardiac dysfunction following myocardial infarction or acute decompensated heart failure [4]. In patients with septic shock, inotropes are recommended when cardiac dysfunction persists despite adequate volume status and arterial blood pressure [5].

Common clinically used inotropic agents include adrenergic agonists like dobutamine and epinephrin, and inhibitors of phosphodiesterase (PDE) like milrinone. Adrenergic agonists provoke slightly different effects on adrenergic receptors and show different pharmacodynamics. Dobutamine is a synthetic catecholamine, derived from isoprenaline, with high affinity to β-adrenergic but low affinity to α-adrenergic receptors [1,6]. Therefore, it increases cardiac output by elevating the heart rate and increasing myocardial inotropy, while systemic and pulmonary vascular resistance remains unchanged [4]. Dobutamine is used in various states of low cardiac output syndromes, sepsis-related myocardial dysfunction, and symptomatic bradycardia [6]. In contrast, epinephrine is a natural catecholamine with high affinity to all adrenergic receptors, and therefore not only increases cardiac output and heart rate but also systemic vascular resistance via the α-adrenergic receptor pathway at higher doses [1,6]. Milrinone inhibits PDE III, provoking the accumulation of cyclic adenosine monophosphate (cAMP). It improves cardiac inotropy and lusitropy, while systemic and pulmonary vascular resistance are significantly lowered, which can be accompanied by arterial hypotension [1]. Adverse side effects of the three inotropic agents include tachycardia, arrhythmia, and cardiac ischemia through increased myocardial oxygen consumption [6].

Respiratory infection and pneumonia can be serious complications during intensive care therapy and in the postoperative period. Indeed, in acute decompensated heart failure, or after non-cardiac and cardiac surgery, postoperative pneumonia is accompanied by significant increases in mortality and morbidity [7,8,9]. Therefore, drugs administered during these periods should be evaluated for their ability to modulate the competence of the respiratory tract to protect the integrity of the airways through the removal of potentially harmful substances in a process known as mucociliary clearance. Mucociliary clearance protects the lung by eliminating from the airways pollutants and colonizing pathogens, therefore preventing infection. Ciliated epithelial cells use outward-directed transportation to prevent the accumulation of debris and potentially harmful substances [10,11]. These cilia are located on the apical side of the respiratory epithelium along the airways, and are constructed by unique structural proteins [10]. Ciliary activity can be measured as ciliary beat frequency (CBF) or particle transport velocity (PTV) [12,13]. The mucociliary clearance function precisely adapts to physiological and pathophysiological conditions through the involvement of many different endogenous and exogenous pathways [11,12]. Inhaled particles, compositions of mucus, electrolytes, and endogenous defensive substances are removed from the lower airways and are subsequently coughed up [11,12]. Ciliary activity is influenced by the sympathetic and parasympathetic nervous systems and their transmitters, norepinephrine and acetylcholine [14,15]. Important intracellular second messengers are cAMP, cyclic guanosine monophosphate, and calcium ions (Ca^2+^), which ultimately induce alterations in CBF [16,17]. Furthermore, ciliary activity is sensitive to local temperature, acid–base balance, humidity, mechanical stress, cytokines released during infection, and paracrine effects mediated by adjacent cells [13,17,18,19,20,21]. Local milieu changes in pathophysiological conditions, like the formation of reactive oxygen species, also influence mucociliary clearance. On the other hand, pathogenic bacterial or fungal components mediate an opposing effect by reducing CBF with subsequent impairment in mucociliary clearance [22,23]. However, mucociliary clearance is also impaired by iatrogenic interventions, like tracheal intubation and mechanical ventilation, which are associated with increased bacterial colonization and pneumonia [24]. Impairment of mucociliary clearance was also found in spontaneously breathing critically ill patients in intensive care units [25]. Impairment of mucociliary clearance may even endure in the postoperative period, after general anesthesia with endotracheal intubation and mechanical ventilation [26].

Recent studies have shown that commonly used antihypotensive drugs, such as a 20:1 mixture of cafedrine/theodrenaline, and norepinephrine, vasopressin, and dopamine all accelerate murine PTV, suggesting the modulation of mucociliary clearance when administered intravenously [27,28]. However, the influence of clinically relevant inotropic agents on the mucociliary clearance remains unknown. Therefore, the aim of this study was to evaluate whether inotropic agents can modulate murine PTV as a measure of mucociliary clearance function. Dobutamine, epinephrine, and milrinone were applied to murine tracheae, and the effects on PTV were evaluated. Specific inhibitory substances were used to reveal the underlying signaling cascades and to gain insight into the modulation of epithelial calcium homeostasis.

## 2. Materials and Methods

### 2.1. Tracheal Preparation and Imaging

All procedures involving animals were conducted in compliance with European legislation for the protection of animals used for scientific purposes and the standards for animal experiments according to German animal welfare law. The experiments were approved by the local committee for animal care of the regional council (Permit number 851_M, Regional Council of Giessen, Giessen, Germany). Tracheal samples were prepared as previously described [13,29]. In brief, male C57BL6J mice weighing 25–35 g (aged 12–15 weeks, purchased from Charles River, Sulzfeld, Germany) were sacrificed. The tracheae were immediately fixed in a dish, and the musculus trachealis was cut open in a longitudinal direction. The respiratory epithelium was visualized in the stage holder of an upright transmission light microscope. A temperature control unit maintained a constant temperature of 30 °C in the center of the buffer solution. Dynabead polymer particles with a mean diameter of 2.8 µm were added to the buffer solution. The tracheal epithelium was then focused between two cartilages in bright-field mode using a 20× water immersion lens, allowing the measurement of PTV indicated by the controlled motion of the Dynabeads along the tracheal epithelium. Drugs and buffer solutions are presented in the Appendix A.

### 2.2. Measurement of PTV

After a 30-min resting period, a subsequent 80-min observation period was established for repeated measurements of PTV performed under the influence of the different drugs. During the first 72 min of the observation period, measurement of PTV was conducted every 3 min. Afterwards, adenosine triphosphate (ATP) was applied to confirm the viability of the tracheal epithelium, leading to a maximal increase in PTV. Measurements of PTV were then performed every 2 min until the end of the experiment at minute 80. At each timepoint, short movie sequences were recorded with high sampling rates using TiLLvisION Imaging software, version 4.0 (Till Photonics, Gräfeling, Germany), as described previously [13,29,30]. Each video sequence consisted of 200 images taken over a period of 16.726 s (one image/83.63 ms) during which approximately 200–400 particle tracks were recorded. Subsequent offline processing was performed using Image Pro Plus analysis software, version 7.0 (Media Cybernetics, Rockville, MD, USA). After background subtraction, the images were converted into grey scale and the formerly dark Dynabeads appeared as bright images. In greyscale, the 12-bit film was reduced to 8-bit for monitoring of individual particle pathways. Particles with less than 15% lateral deviation were included in the analysis. From these measurements, the average PTV was calculated for each timepoint.

### 2.3. Statistical Analysis

Tracheal preparations and PTV measurements were only included in the statistical analyses when a clear response to the application of ATP was detected at the end of the experiments. The absolute PTV value after the resting time and prior to the observation period was standardized to 100%. PTV values at distinct timepoints are presented as mean and standard error of the mean (SEM), while peak plateau PTV values of each experimental group are shown as the median with interquartile range. Median effective concentrations (EC_50_) were calculated using global nonlinear regression. Two-way analysis of variance (ANOVA) for repeated measurement models, including the experimental groups of each analyzed substance, were created, and inter-group differences over time were evaluated using post hoc Bonferroni’s test. Statistical analysis was performed with R statistics, version 4.0.4 (www.r-project.org), and GraphPad PRISM was utilized for figure creation (version 9.5.0, GraphPad Software, La Jolla, CA, USA). In general, two-tailed values of *p* < 0.05 were considered statistically significant.

## 3. Results

### 3.1. Drug Dose Dependency of Murine PTV

**Figure 1:**
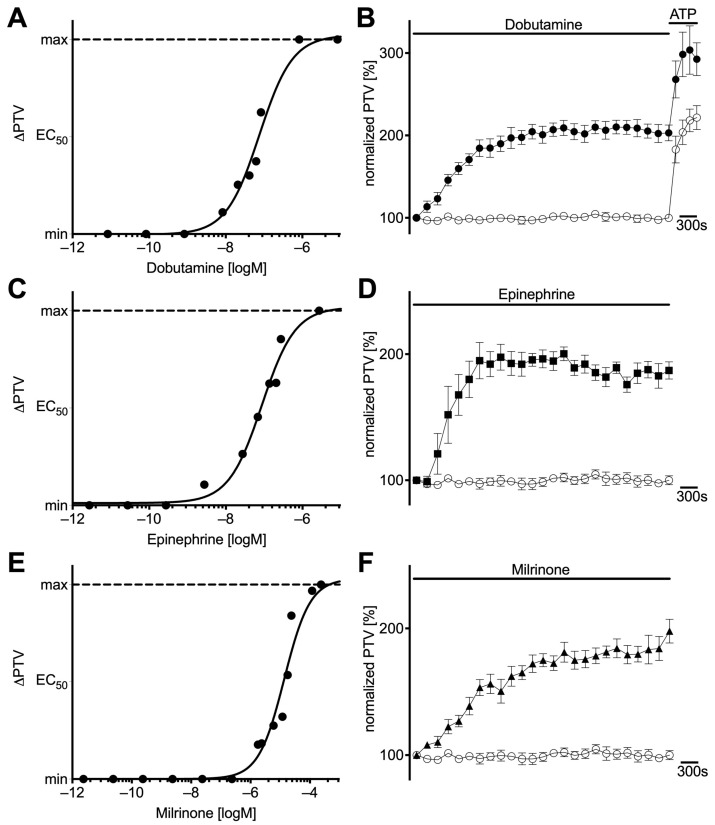
Dobutamine, epinephrine, and milrinone increased particle transport velocity (PTV) in a concentration-dependent manner. Concentration–response relationships of (**A**) dobutamine (n = 10), (**C**) epinephrine (n = 10), and (**E**) milrinone (n = 14) described by global nonlinear regression (line). A persistent increase in murine PTV was observed after the application of median effective concentrations of (**B**) dobutamine (●, 75.7 nM), (**D**) epinephrine (◼, 87.0 nM), and (**F**) milrinone (▲, 13.7 µM). PTV remained constant around its baseline value in control experiments (○). ⊥ standard error of the mean.

Dobutamine, epinephrine, and milrinone were administered to murine tracheae in increasing concentrations, and concentration–response curves were established (Figure 1A,C,E). The calculated EC_50_ were 75.7 nM for dobutamine, 87.0 nM for epinephrine, and 13.7 µM for milrinone. The PTV remained constant around its baseline under control conditions (99 [93−106] %, n = 7) (Figure 1B,D,F). When the EC_50_ of each substance was administered, the PTV significantly increased throughout the whole observation period and substance-specific plateaus were reached (dobutamine 191 [181−222] % (n = 6), epinephrine 195 [181−207] % (n = 5), milrinone 174 [166−185] % (n = 5), each *p* < 0.001) (Figure 1B,D,F). An additional steep increase following the administration of ATP confirmed the vitality of the tracheal epithelium at the end of each experiment. This is demonstrated by the example in Figure 1B.

### 3.2. Influence of Adrenergic Receptors

**Figure 2:**
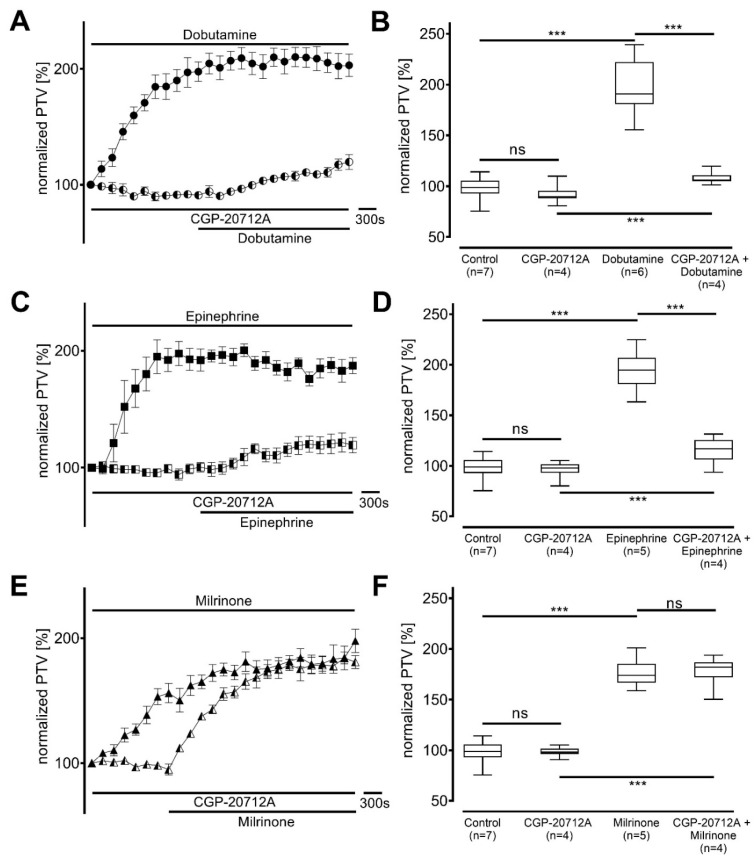
Increase in particle transport velocity (PTV) with dobutamine and epinephrine, but not milrinone, largely dependent on β_1_-adrenergic receptors. (**A**,**B**) Only a small PTV increase was observed with dobutamine and the selective β_1_-adrenergic receptor inhibitor CGP-20712A (0.1 µM). (**C**,**D**) Similar dynamics occurred when epinephrine was administered. (**E**,**F**) PTV equally increased when milrinone was applied with CGP-20712A. *** *p* < 0.001, ns: not significant. ● dobutamine (n = 6), ◐ dobutamine + CGP-20712A (n = 4), ◼ epinephrine (n = 5), ◧ epinephrine + CGP-20712A (n = 4), ▲ milrinone (n = 5), ◭ milrinone + CGP-20712A (n = 4), ⊥ standard error of the mean, box and whisker plots indicate median, interquartile range (box), minimum and maximum (whiskers).

β_1_-Adrenergic receptors were selectively inhibited with CGP20712A (Figure 2). CGP20712A alone did not alter the PTV compared to the control experiments (dobutamine 90 [88−95] %, *p* = 0.523; epinephrine 98 [93−102] %, *p* > 0.999; milrinone 98 [96−102] %, *p* > 0.999; each n = 4). The PTV increased slightly, but with statistical significance, after the application of dobutamine (106 [105−111] %, *p* < 0.001) (Figure 2A,B) and epinephrine (117 [106−126] %, *p* < 0.001) (Figure 2C,D). However, the plateaus reached were clearly lower than those observed in the absence of CGP20712A (each *p* < 0.001). In contrast, a strong increase in PTV was measured after administration of milrinone (182 [172−187] %, *p* < 0.001) (Figure 2E,F) despite the presence of CGP20712A, and the plateau was comparable to that of milrinone with no inhibitor (*p* > 0.999).

**Figure 3:**
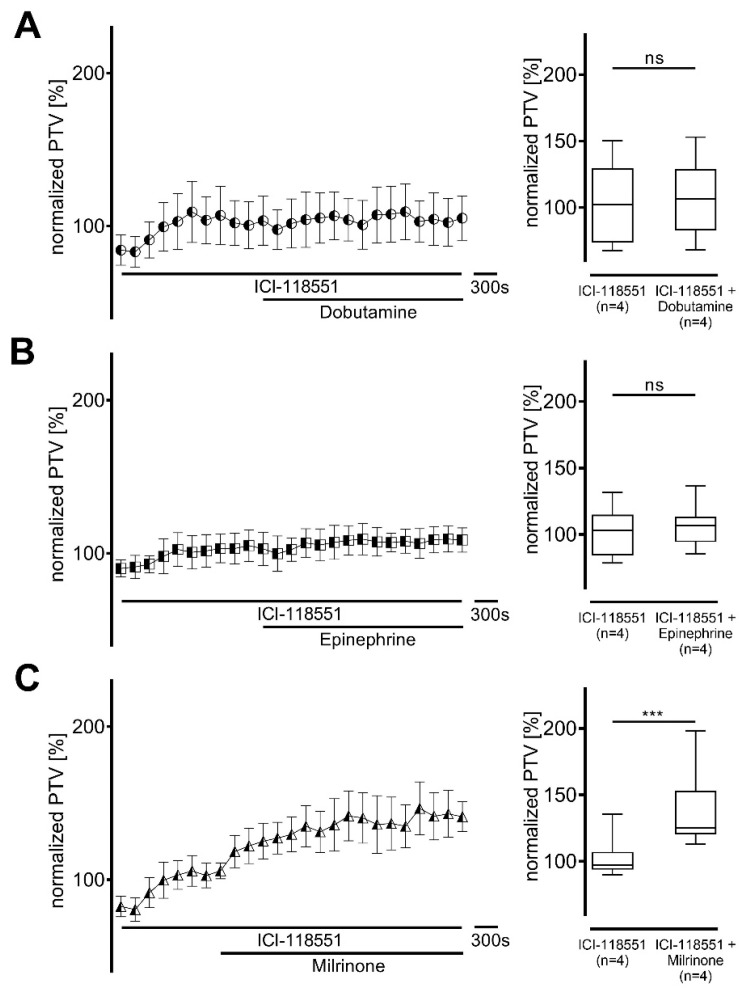
Alteration in particle transport velocity (PTV) following administration of dobutamine and epinephrine, but not milrinone, is dependent on β-adrenergic receptor activation. β-Adrenergic receptors were non-selectively inhibited by ICI-118551 (100 µM). No increase in PTV was observed with (**A**) dobutamine or (**B**) epinephrine, but PTV increased significantly with (**C**) milrinone. PTV was normalized to the baseline with ICI-118551. *** *p* < 0.001, ns: not significant, ◐ dobutamine + ICI-118551 (n = 4), ◧ epinephrine + ICI-118551 (n = 4), ◭ milrinone + ICI-118551 (n = 4), ⊥ standard error of the mean, box and whisker plots indicate median, interquartile range (box), minimum and maximum (whiskers).

In the next experimental series, β-adrenergic receptors were non-selectively inhibited by ICI-118551. Since alterations in the PTV baseline were observed in the presence of ICI-118551, the baseline was normalized to the median PTV value during incubation with ICI-118551 (dobutamine 102 [74−130] %; epinephrine 103 [84−115] %; milrinone 97 [94−107] %, each n = 4). Subsequent changes in PTV after administration of the inotropic agents were then evaluated. No significant increase in PTV was observed when dobutamine (107 [83−129] %, *p* > 0.999; Figure 3A) or epinephrine (107 [94−114] %, *p* = 0.963; Figure 3B) were administered. However, the increase in PTV was maintained under the influence of milrinone (125 [120−153] %, *p* < 0.001; Figure 3C).

### 3.3. Influence of Key Signal-Transduction Enzymes

**Figure 4:**
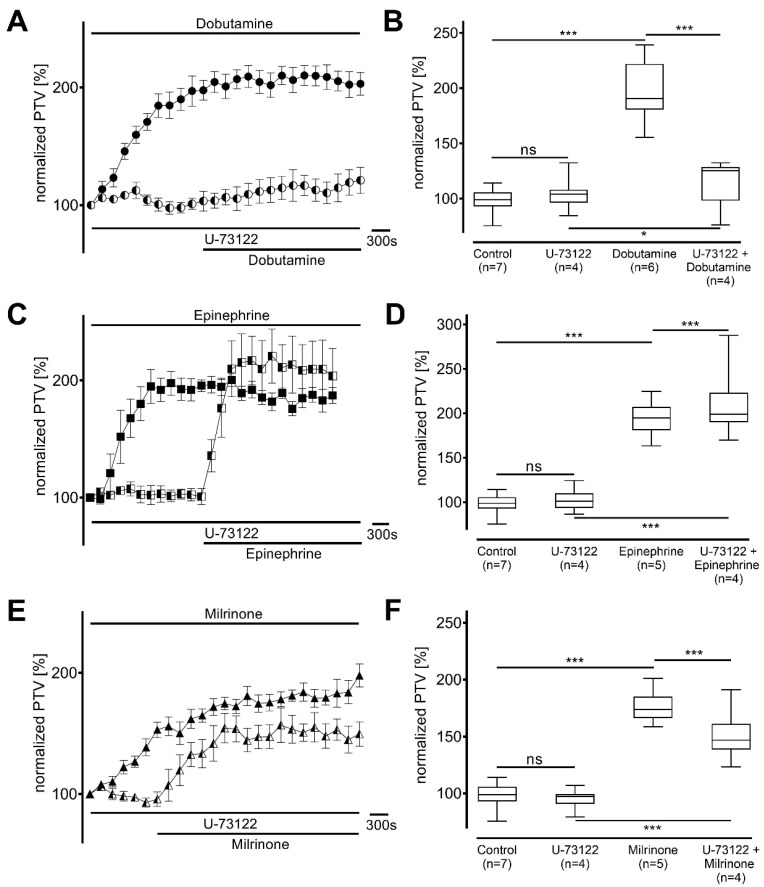
Increase in particle transport velocity (PTV) with dobutamine, but not epinephrine and milrinone, largely dependent on phospholipase C (PLC). Baseline PTV did not change when selective PLC inhibitor U-73122 (2.5 µM) was applied. (**A**,**B**) PTV increase with dobutamine was mostly inhibited under PLC inhibition. (**C**,**D**) Epinephrine caused an increase in PTV equal to PTV without PLC inhibition. (**E**,**F**) Maximum PTV still significantly increased, but was reduced with milrinone. * *p* < 0.05, *** *p* < 0.001, ns: not significant. ● dobutamine (n = 6), ◐ dobutamine + U-73122 (n = 4), ◼ epinephrine (n = 5), ◧ epinephrine + U-73122 (n = 4), ▲ milrinone (n = 5), ◭ milrinone + U-73122 (n = 4), ⊥ standard error of the mean, box and whisker plots indicate median, interquartile range (box), minimum and maximum (whiskers).

The influence of phospholipase C (PLC) and PKA was analyzed in the next experimental series. PLC was inhibited with U-73122, which did not alter the PTV alone (dobutamine: 104 [96−108] %; epinephrine: 101 [94−110] %; milrinone: 97 [90−99] %; each *p* > 0.999 and n = 4; Figure 4). PTV was only barely increased by dobutamine during PLC inhibition (125 [98−128] %, *p* = 0.019; Figure 4A,B). In contrast, the PTV was affected by epinephrine in the presence of U-73122 (199 [190−223] %, *p* < 0.001; Figure 4C,D), provoking an even higher increase in PTV than without PLC inhibition. Milrinone was able to increase the PTV significantly (147 [139−161] %, *p* < 0.001; Figure 4E,F), although the maximum amplitude was lower than the plateau without PLC inhibition (*p* < 0.001).

**Figure 5:**
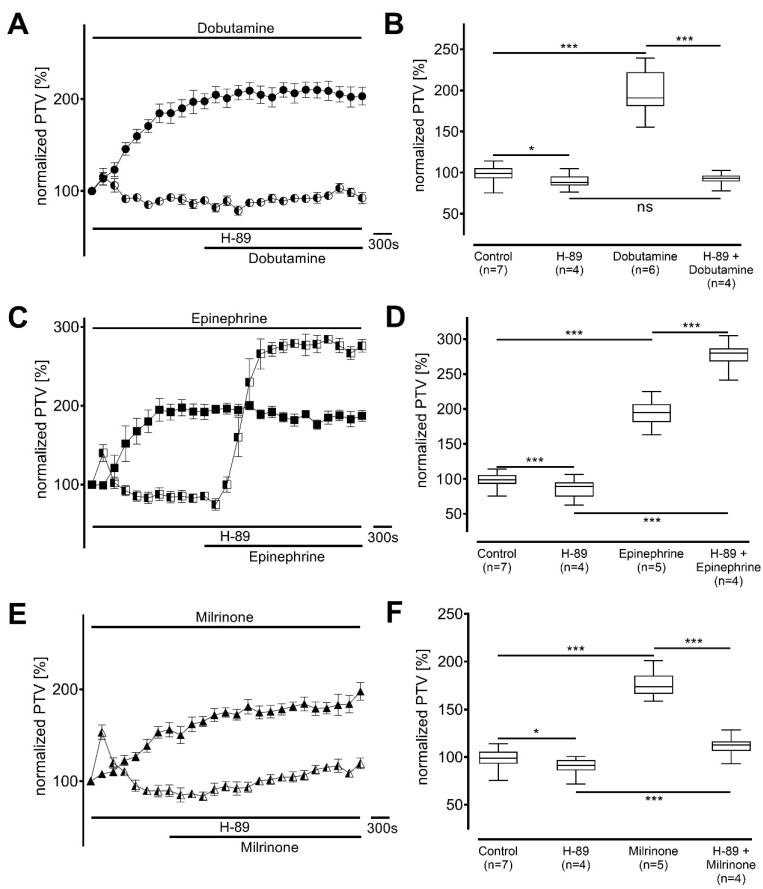
Protein kinase A (PKA) significantly contributes to the particle transport velocity (PTV) increase with dobutamine and milrinone, but not epinephrine. Baseline PTV decreased when selective PKA inhibitor H-89 (10 µM) was applied. (**A**,**B**) PTV did not increase when dobutamine was applied. (**C**,**D**) Epinephrine caused a significant increase in PTV. (**E**,**F**) PTV increased only modestly with milrinone resulting in values around the initial baseline, * *p* < 0.05, *** *p* < 0.001, ns: not significant. ● dobutamine (n = 6), ◐ dobutamine + H-89 (n = 4), ◼ epinephrine (n = 5), ◧ epinephrine + H-89 (n = 4), ▲ milrinone (n = 5), ◭ milrinone + H-89 (n = 4), ⊥ standard error of the mean, box and whisker plots indicate median, interquartile range (box), minimum and maximum (whiskers).

When the PKA was blocked using H-89, baseline PTV was significantly reduced (dobutamine: 88 [84−96] %, *p* < 0.030; epinephrine: 89 [75−95] %, *p* < 0.001; milrinone: 91 [86−97] %, *p* = 0.041; each n = 4; Figure 5), and no further alteration in PTV was observed when dobutamine was added (93 [89−96] %, *p* > 0.999; Figure 5A,B). In contrast, again, epinephrine still provoked a significant increase in PTV (280 [268−287] %, *p* < 0.001, Figure 5C,D). Milrinone was able to significantly accelerate PTV (113 [106−117] %, *p* < 0.001, Figure 5E,F) but the plateau was significantly lower than that measured without PKA inhibition (*p* < 0.001).

### 3.4. Evaluation of Extracellular Ca^2+^, Internal Calcium Stores, and Ca^2+^-Releasing Channels

**Figure 6:**
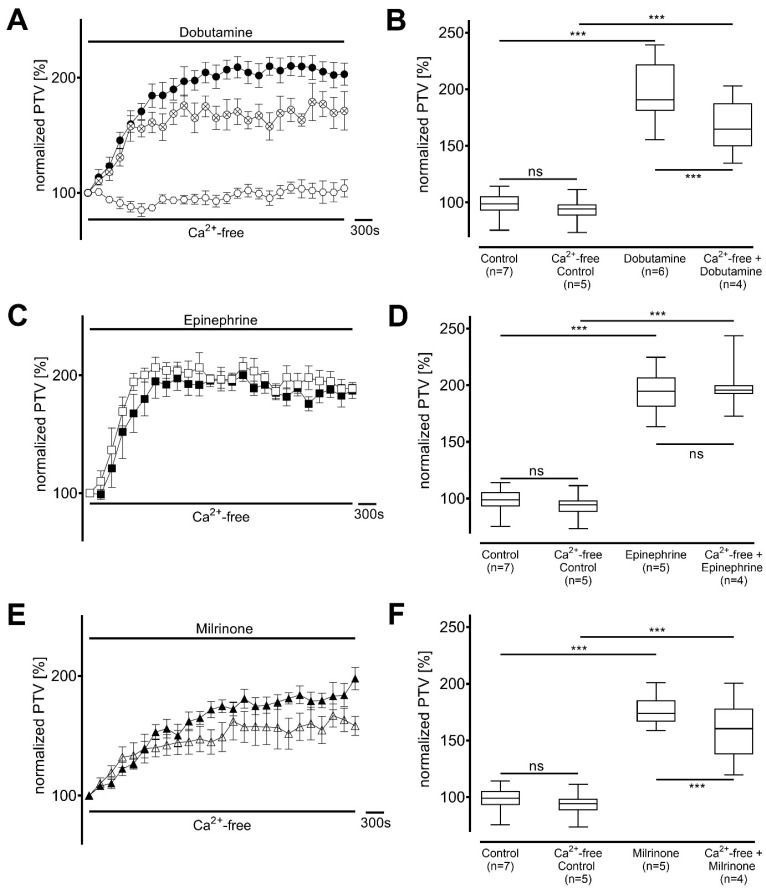
Increase in particle transport velocity (PTV) with milrinone and dobutamine is slightly reduced when extracellular Ca^2+^ entry is precluded, whereas the PTV with epinephrine remained unchanged. PTV was measured in Ca^2+^-free buffer solution with (**A**,**B**) dobutamine, (**C**,**D**) epinephrine and (**E**,**F**) milrinone. *** *p* < 0.001, ns: not significant. ○ Ca^2+^-free control, ● dobutamine (n = 6), ⊗ dobutamine in Ca^2+^-free buffer (n = 4), ◼ epinephrine (n = 5), □ epinephrine in Ca^2+^-free buffer (n = 4), ▲ milrinone (n = 5), △ milrinone in Ca^2+^-free buffer (n = 4), ⊥ standard error of the mean, box and whisker plots indicate median, interquartile range (box), minimum and maximum (whiskers).

The extent of extracellular Ca^2+^ entry was evaluated when the inotropic agents were administered to tracheae in Ca^2+^-free buffer solution, which precluded extracellular Ca^2+^ entry. Under control conditions, PTV remained constant throughout the observation period (94 [88−98] %, n = 5, Figure 6A). Dobutamine was still able to significantly increase the PTV (165 [149−188] %, *p* < 0.001, n = 4; Figure 6A,B), but maximum amplitude was significantly lower than the plateau observed in Ca^2+^-containing buffer (*p* < 0.001). In contrast, the dynamics of epinephrine were highly comparable, and the plateau observed following epinephrine administration (196 [192−200] %, *p* = 0.228, n = 4; Figure 6C,D) did not differ from that seen in Ca^2+^-containing buffer. When milrinone was administered in Ca^2+^-free buffer, a slight reduction in maximum PTV was observed (160 [138−178] %, *p* < 0.001, n = 4; Figure 6E,F).

**Figure 7:**
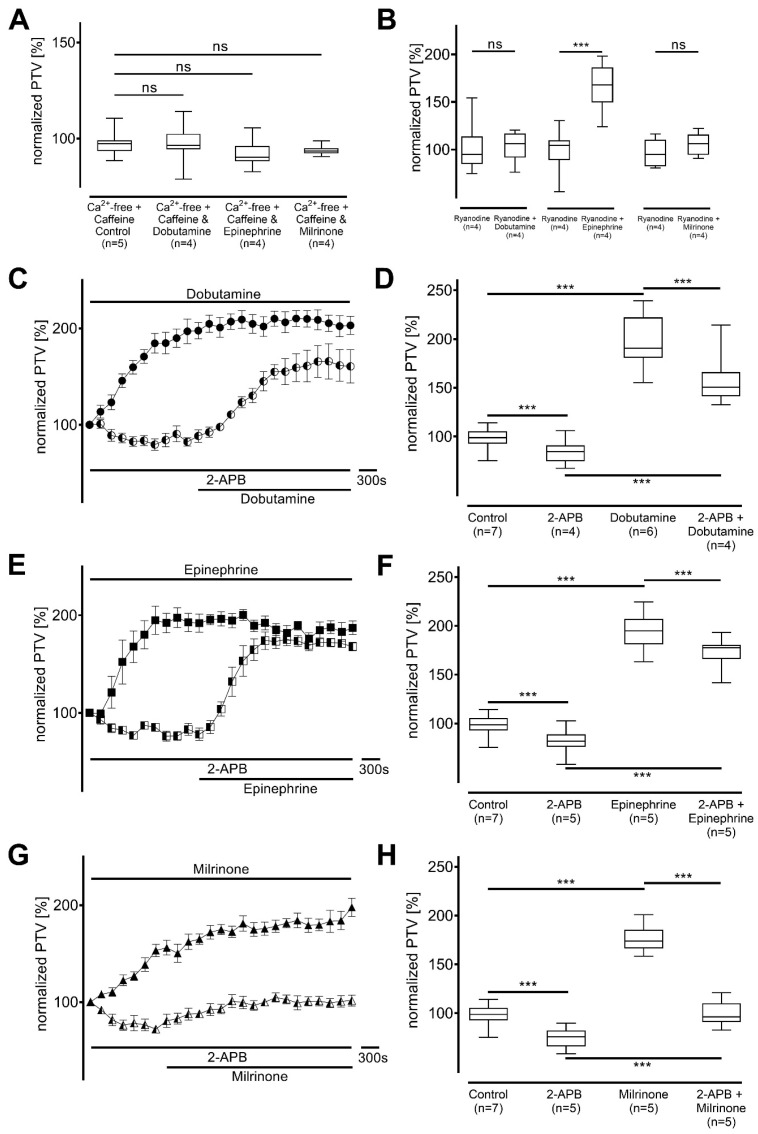
Release of intracellular Ca^2+^ differs with dobutamine, epinephrine, and milrinone. (**A**) Increase in particle transport velocity (PTV) is fully dependent on intracellular Ca^2+^ stores, and no increase in PTV was observed in Ca^2+^-free buffer after intracellular stores were depleted by caffeine (30 mM). (**B**) When ryanodine receptors were inhibited with ryanodine (40 µM), only epinephrine was able to increase PTV, while PTV was normalized to baseline in the presence of ryanodine. When IP_3_ receptors were selectively inhibited by 2-aminoethoxydiphenylborane (2-APB), (**C**,**D**) dobutamine, and (**E**,**F**) epinephrine only slightly reduced maximum PTV values, while (**G**,**H**) milrinone only modestly increased PTV. *** *p* < 0.001, ns: not significant. ● dobutamine (n = 6), ◐ dobutamine + 2-APB (n = 4), ◼ epinephrine (n = 5), ◧ epinephrine + 2-APB (n = 5), ▲ milrinone (n = 5), ◭ milrinone + 2-APB (n = 5), ⊥ standard error of the mean, box and whisker plots indicate median, interquartile range (box), minimum and maximum (whiskers).

Based on these results, internal Ca^2+^-stores and their key Ca^2+^-releasing channels were evaluated in the next experiments. Caffeine sensitive stores, comprised mainly by the endoplasmic reticulum (ER), were depleted by caffeine (97 [93−99] %, n = 5), and subsequent administration of dobutamine (97 [94−102] %), epinephrine (90 [86−96] %), and milrinone (93 [92−95] %) caused no alteration to the PTV (each *p* > 0.999 and n = 4) (Figure 7A).

Ryanodine receptors were blocked by ryanodine, and alterations in the PTV baseline provoked by ryanodine were normalized to the median PTV during the incubation of ryanodine (each n = 4). Ryanodine receptor inhibition vanished any change in PTV following the administration of dobutamine (95 [85−114] % vs. 106 [92−116] %, *p* > 0.999) and milrinone (95 [83−111] % vs. 107 [95−116] %, *p* = 0.275). However, epinephrine was still able to provoke a significant increase in PTV (105 [90−111] % vs. 169 [150−187] %, *p* < 0.001; Figure 7B).

Subsequent experiments focused on the role of inositol triphosphate (IP_3_) receptors, in which the tracheae were incubated with the inhibitor 2-APB (Figure 7E–H). A significant decrease in baseline PTV was observed during the incubation with 2-APB (dobutamine: 85 [75−91] %; epinephrine: 82 [76−89] %; milrinone: 76 [66−82] %; each *p* < 0.001 and n = 4). PTV significantly increased when dobutamine (151 [141−166] %, *p* < 0.001; Figure 7C,D) or epinephrine (177 [166−181] %, *p* < 0.001; Figure 7E,F) were added. However, milrinone only slightly increased the PTV to values approximately those of control experiments (96 [91−110] %, *s* < 0.001; Figure 7G,H).

## 4. Discussion

Our results show that the frequently used inotropic agents dobutamine, epinephrine, and milrinone have specific effects on PTV, suggesting changes in the mucociliary clearance. Concentration response curves could be derived for all agents, indicating specific effects at different sites of the complex signal transduction cascades that modulate cilia kinetics. All analyzed agents improved ciliary transport movement, which is ultimately triggered by intracellular Ca^2+^ release. However, different signal transduction cascades provoking Ca^2+^ release are involved depending on the specific properties of the individual substances.

In our study, according to the known receptor affinities of dobutamine and epinephrine, both agents primarily acted on adrenergic receptors. Isolated β_1_-adrenergic receptor inhibition was unable to completely prevent the effects of dobutamine and epinephrine, although the observed increases in PTV were small compared to those without β_1_-adrenergic receptor inhibition. Subsequent experiments revealed that unselective β-adrenergic blockage could completely prevent the increase in PTV following dobutamine or epinephrine treatment. Therefore, we conclude that both agents primarily act on β_1_-adrenergic receptors, but some effects were also unselectively mediated on other β-adrenergic receptors. Although both β-adrenergic receptor subtypes are generally known to be expressed in the respiratory tract, β_1_-adrenergic receptors constitute only a small proportion of those in the bronchial tree [31]. However, the abolished increase in PTV with epinephrine under unselective β-adrenergic receptor inhibition confirms previous results that α-adrenergic receptors may not be involved in the regulation of mucociliary clearance. Indeed, only the α1D subunit was found in the isolated murine respiratory epithelium [22].

Interestingly, dobutamine and epinephrine showed different kinetics when key downstream signal transduction cascades were analyzed following adrenergic receptor activation. While epinephrine still provoked significant effects on the PTV when the PKA or PLC pathways were inhibited, dobutamine failed to increase PTV when PKA and ryanodine receptors were inhibited. However, PLC was seen to exert some influence because PTV was markedly reduced in the presence of U-73122. Therefore, the reasons for epinephrine and dobutamine showing different intracellular effects, despite both agents inducing comparable effects at the β-adrenergic receptors, require discussion. In general, the PKA pathway represents the classical β_1_-adrenergic receptor pathway, activated by cAMP after induction of the α subunit of the stimulating G protein. However, non-canonical signal pathways leading to Ca^2+^ release have also been described. For example, the PLC pathway can be activated directly by cAMP or by the βγ subunits of β-adrenergic receptors [32,33]. The alternative downstream pathways then ultimately release Ca^2+^ via IP_3_ receptors [34].

Therefore, our experiments with downstream pathway inhibitors were unable to prevent the increase in PTV when epinephrine was administered because only single inhibitors were used in each experimental series. β-Adrenergic receptor activation was strong enough to cause sufficient Ca^2+^ release to raise PTV to levels comparable to those without specific inhibitory substances. In contrast, dobutamine showed more classical behavior by involving primarily the PKA and ryanodine receptors, although some influence of PLC was also observed. However, our experiments with 2-APB and dobutamine indicated no influence of IP_3_ receptors on PTV.

We conclude that the different effects of epinephrine and dobutamine have their origin at the adrenergic receptor binding sites. First, different receptor affinities might be involved, because dobutamine acts on β_1_- and β_2_-adrenergic receptors in a 3:1 ratio, while epinephrine acts as an agonist on all adrenergic receptors [6]. Furthermore, epinephrine is the natural agonist of the adrenergic receptors, with the highest affinity and efficacy of all adrenergic receptor agonists. However, our experiments were unable to capture different effects of epinephrine and dobutamine at the murine epithelium when adrenergic receptors were inhibited. This could be explained by biased agonism, whereby different agents binding to the same receptor provoke specific receptor conformation changes, activating distinct downstream pathways. Biased agonism is a well-known effect at adrenergic receptors [35] and might explain the PKA-selective pathway of dobutamine, and the broader effects of the natural agonist epinephrine which could not be inhibited by blocking only one single pathway because the non-inhibited pathways might be strong enough to provoke sufficient release of Ca^2+^. Dynamics comparable to those of epinephrine have been recently observed for norepinephrine in the murine tracheal epithelium, effects also provoked by adrenergic receptor activation [27]. Different downstream cascades in the murine tracheal epithelium were also observed for other β-adrenergic agonists, such as cafedrine and theodrenaline, effects that were blocked neither by PKA nor PLC inhibition. However, in contrast to our study with inotropic agents, IP_3_ receptors were still found to release Ca^2+^, ultimately increasing PTV [30].

In comparison to dobutamine and epinephrine, milrinone caused different effects, although PTV also significantly increased with higher concentrations, as illustrated by the right-shifted concentration–response curve. Since milrinone intracellularly inhibits PDE III, thus increasing cAMP concentrations, no effects of adrenergic receptor blockage were observed when the PTV was raised by milrinone. Although this finding might be obvious, it confirms that adrenergic receptor activation with a subsequent increase in cAMP concentration might not be pivotally involved in regulating the baseline PTV in isolated tracheae, despite the observation that neither inhibitory agent lowered the baseline PTV. Interestingly, our experiments reveal that the effects on the murine PTV induced by milrinone through increased cAMP concentrations are subsequently modulated by both PKA and PLC signal pathways. On the one hand, the increase in PTV did not completely vanish when PKA was inhibited because a small and delayed, but nonetheless significant, increase in PTV was observed. On the other hand, the increase in PTV was stronger, but nonetheless significantly reduced, in the presence of PLC inhibitor U-73122. Therefore, we conclude that the PLC pathway is also activated by cAMP under the presence of milrinone, as discussed above. Furthermore, this crosslink between cAMP and PLC might become specifically relevant because an accumulation of cAMP induced by milrinone might accelerate the induction of PLC more than under physiological conditions.

Consequently, Ca^2+^ release from the ER under the presence of milrinone is modulated by both ryanodine and IP_3_ receptors. PTV still increased when IP_3_ receptors were inhibited, but to a much lesser extent. Only a small, numerically but not statistically significant, increase was measured when ryanodine receptors were blocked. Therefore, these results might reflect the relevance of both PKA and PLC pathways to the increase in murine PTV under the influence of milrinone. However, ryanodine and IP_3_ receptor coactivation must be discussed, especially in light of the result that no significant increase in PTV was observed when ryanodine receptors were inhibited. Therefore, the activation of IP_3_ receptors through ryanodine receptor-induced Ca^2+^ release, generally known as Ca^2+^-induced Ca^2+^ release (CICR), must also be considered [36]. However, if CICR explains the results in the experiments under IP_3_ receptor inhibition, it should be generally observed with other substances like dobutamine, as in our experiments. Since the effects were only observed under the influence of milrinone, we conclude that the additional activation of PLC by cAMP is primarily responsible for the involvement of IP_3_ receptors.

When extracellular Ca^2+^ entry was precluded using Ca^2+^-free buffer solution, slight differences in maximum PTV amplitude were observed with dobutamine and milrinone. However, the PTV increase provoked by epinephrine was identical to the increase seen in Ca^2+^-containing buffer. Since the amplitudes were only marginally reduced with dobutamine and milrinone, we conclude that extracellular Ca^2+^ may not be clinically relevant when inotropic agents alter the ciliary activity in murine tracheal epithelium. Different results have been observed when other antihypotensive drugs were evaluated in the same experimental setting. Norepinephrine, as a precursor of epinephrine, and vasopressin did show markedly reduced PTV increase, while dopamine, another precursor of epinephrine, showed comparable PTV dynamics [27]. Our results also confirm that the observed extracellular Ca^2+^ entry is secondary to intracellular Ca^2+^ release, which is primarily derived from caffeine-sensitive stores, as shown in experiments with caffeine in which any increase in PTV vanished. As in excitable cells, Ca^2+^ is a key second messenger in non-excitable cells [37]. Therefore, the extracellular Ca^2+^ entry evaluated in Ca^2+^-free buffer should prevent store-operated Ca^2+^ entry (SOCE) [38]. In general, the inhibition of SOCE is challenging because it is not provided by a single path but is modulated by multiple targets and channels, such as the calcium release-activated calcium channel protein ORAI 1 [39]. These channels open after the activation of stromal interaction molecules located in the transmembrane area of the ER that sense decreased Ca^2+^ concentration [40]. Hence, it is difficult to conduct experiments in which differential signal transduction pathways of these transmembrane receptors and proteins are inhibited. Therefore, our experiments focused on screening for the relevance of overall extracellular Ca^2+^ entry using a Ca^2+^-free buffer solution. Future studies could focus on evaluating the value of SOCE in tracheal epithelial cells, although inotropic agents may not be suitable substances to evaluate SOCE, as we have shown in our experiments with Ca^2+^-free buffer.

## 5. Limitations

Several limitations of our experimental series must be acknowledged. First, we used murine tissues. While this is a valid approach to basic research questions, transferability to human tissues and the assessment of clinical significance must be performed with caution. Unfortunately, in vivo experimental approaches in critically ill patients who receive the inotropes analyzed in our experiments are highly limited not only due to the many potential confounding factors during intensive care but also due to limited methodologies to measure mucociliary clearance function in clinical scenarios. Second, isolated tracheae were studied, so integrity of the tissues was at least partly disrupted, which might have allowed the atypical entrance of the administered drugs. Furthermore, the drugs were administered within the buffer where the tracheae were placed. Therefore, the drugs did not work via pulmonary capillary perfusion but via direct administration to the basal and apical side. However, epinephrine and milrinone are also administered by inhalation in clinical practice, and the apical layer of the murine tracheal epithelium was preserved, as demonstrated by the preserved targeted motion of cilia transport. As we used tracheal tissues, transfer to the function of the lower respiratory tract and other parts of the respiratory system must also be conducted with caution. However, there are no data that suggest other mechanisms apply for the ciliary-bearing cells of the lower respiratory tract. Third, local concentrations of the analyzed inotropes in clinical practice are unknown. Concentrations might vary significantly between different tissues and at different timepoints. For example, local concentrations of central-venously administered drugs might be temporarily higher in the pulmonary vascular bed compared to concentrations after subsequent systemic circulation. Finally, a limited sample size was studied in our experiments due to rigid animal welfare regulations that obliged us to use the smallest possible sample size. Nonetheless, the observed effects were strong and withstand the statistical analyses with ANOVA for repeated measurement models.

## 6. Conclusions

In conclusion, the inotropic agents dobutamine, epinephrine, and milrinone increase murine PTV in a concentration-dependent manner via substance specific pathways. All agents ultimately release Ca^2+^ from intracellular calcium stores which is, at least for dobutamine and milrinone, followed by extracellular Ca^2+^ influx to achieve the maximal alteration in PTV. Further experimental series should evaluate the differences observed between both adrenergic agonists, dobutamine and epinephrine. Further research should also be conducted with human tissues and in clinical scenarios to evaluate the clinical relevance of these results.

## Data Availability

The raw data supporting the conclusions of this article will be made available by the authors on request.

## Abbreviations

The following abbreviations are used in this manuscript:

ANOVA	analysis of variance
ATP	adenosine triphosphate
cAMP	cyclic adenosine monophosphate
Ca^2+^	calcium ions
CBF	ciliary beat frequency
CICR	Ca^2+^-induced Ca^2+^ release
EC50	median effective concentration
ER	endoplasmic reticulum
IP_3_	inositol trisphosphate
PDE	phosphodiesterase
PKA	protein kinase A
PLC	phospholipase C
PTV	particle transport velocity
SEM	standard error of the mean
SOCE	store-operated Ca^2+^ entry

## Appendix A

Drugs and buffer solutions. All preparations and experiments were performed in 4-(2-hydroxyethyl)-1-piperazine ethanesulfonic acid (HEPES) solution consisting of 10 mM HEPES, 5.6 mM KCl, 2.2 mM CaCl_2_, 11 mM glucose, 136 mM NaCl, and 2.2 mM MgCl_2_. NaOH was used to adjust the pH to 7.4 at 30 °C. To achieve experiments in Ca^2+^-free solutions, CaCl_2_ was substituted with 1 mM ethylene glycol tetraacetic acid. The following drugs were applied during the experiments: 2-aminoethoxydiphenylborane (2-APB, 40 µM diluted in 8 µL dimethyl sulfoxide (DMSO), TOCRIS Bioscience, Bristol, UK), adenosine triphosphate (ATP, 150 µM in 6 µL H_2_O, Sigma-Aldrich, St. Louis, MO, USA), caffeine (30 mM in 600 µL HEPES, Roth, Karlsruhe, Germany), CGP20712A (0.1 µM diluted in 2 µL H_2_O or 100 µM diluted in 20 µL H_2_O, TOCRIS Bioscience, Bristol, UK), dobutamine (75.7 nM in 100 µL H_2_O, Fresenius Kabi Deutschland GmbH, Bad Homburg v.d.H, Germany), epinephrine (87.0 nM in 100 µL H_2_O, Cheplapharma, Greifswald, Germany), H-89 (10 µM diluted in 20 µL DMSO, Sigma-Aldrich, St. Louis, MO, USA), ICI-118551 (100 µM in 20 µL H_2_O, Merck KGaA, Darmstadt, Germany) milrinone (13.7 µM in 100 µL H_2_O, Hikma Pharmaceuticals, London, UK), ryanodine (40 µM in 32 µL DMSO, TOCRIS Bioscience, Bristol, UK), U-73122 (2.5 µM diluted in 2 µL DMSO, Enzo Life Sciences, Farmingdale, NY, USA). The reported drug concentrations were achieved during the experiments after applying the stock solution to the buffer solution in the recording chamber.
